# Risk Factors for Renal Cell Cancer in a Japanese Population

**DOI:** 10.4137/cmo.s2669

**Published:** 2009-06-15

**Authors:** Masakazu Washio, Mitsuru Mori

**Affiliations:** 1 Department of Community Health and Clinical Epidemiology, St. Mary’s College, Kurume City, Fukuoka, 830-8558, Japan.; 2 Department of Public Health, Sapporo Medical University School of Medicine, Sapporo City, Hokkaido, 060-8556, Japan.

**Keywords:** renal cell cancer, risk factor, Japan

## Abstract

The incidence of renal cell cancer has been increasing worldwide. Although the incidence of renal cell cancer in Japan is lower than the rates in the other industrialized countries, there is no doubt that it is increasing. In this paper, we would like to introduce the result of our studies, which evaluate the risk factors for renal cell cancer in Japan. Hypertension, diabetes mellitus, kidney diseases, fondness for fatty food and black tea showed an increased risk of renal cell carcinoma while an intake of starchy roots such as taro, sweet potato and potato reduced the risk of renal cell carcinoma. In Japan, however, drinking black tea may be a surrogate for westernized dietary habits while eating starchy roots may be a surrogate for traditional Japanese dietary habits. Further studies may be needed to evaluate risk factors for renal cell cancer because the number of renal cancer cases was small in our studies in spite of a large population-based cohort study.

## Introduction

Kidney cancer includes both cancer of the renal parenchyma (i.e. renal cell cancer) and cancer of the pelvis (i.e. transitional cell cancer). In adults, 85%–90% of kidney cancer cases are renal cell cancer.^1^ Renal cell cancer accounts for 2%–3% of all malignancies in western countries^2^–^5^ and 1%–2% in Japan.^5^,^6^ The incidence of renal cell cancer is high in Western, and Northern Europe and North America while it is low in Asia.^1^–^3^,^5^ However, the incidence of renal cell cancer is higher in Japanese Americans than in native Japanese.^5^ These findings suggest that the environmental factors such as life-style factors may play a role in the development of renal cell cancer.

The incidence of and mortality from renal cell cancer have been increasing in recent years in Japan.^6^ Westernization of the lifestyle (e.g. westernization of eating habits, the spread of privately-owned cars and household electric appliances as well as agricultural mechanization) may increase the incidence of renal cell cancer. According to the westernization of dietary habits, it decreased to eat traditional Japanese foods (i.e. eating a lot of rice with salty food such as salty grilled fish, salty pickles and soybean paste soup, and drinking green tea), and it increased to take westernized foods (i.e. eating animal protein and fat such as breaded pork cutlet and beefsteak, and drinking coffee or black tea). High-fat and high-protein diet is reported to increase the risk of renal cell cancer.^7^ Low physical activity is also reported to play a role in the development of renal cell cancer.^8^,^9^ Many epidemiologists have reported risk factors for renal cell cancer in western countries.^3^ Even in textbooks of renal cell cancer written in Japanese,^6^ however, we have to get knowledge about the risk factors for renal cell cancer from studies in western countries^1^–^3^ because we can get only a little information about renal cell cancer in the census-based cohort study on the relationship between lifestyle and mortality in Japan.^10^

Therefore, we evaluated the risk factors for renal cell cancer in a large population-based cohort study in Japan (JACC study),^11^ which has followed up for more than 1 million-person years. Briefly, the original study population consisted of 46,465 men and 64,327 women aged 40 to 79 years in 45 areas of 19 prefectures in Japan. Enrolment began in 1988 and continued 1990. Most subjects were recruited from the general population when undergoing routine health checks in the municipalities. Most study subjects (86.3%) were followed up for mortality until the end of 2003, except in a few areas where the follow-up periods were until the end of 1999. However, incident cancer were ascertained only 24 study area (out of 45) in which cancer registries were available, where the mortality-incident rate deviated from 0.31 to 0.61 in men and from 0.15 to 0.53 in women.^12^

In this paper, we would like to introduce the result of our studies,^13^–^15^ which evaluate the risk factors for renal cell cancer in a Japanese population.

## Descriptive Epidemiology

The incidence of renal cell cancer has been increasing worldwide.^1^ The rapidly increasing incidence of renal cell carcinoma may be partly explained by the rising numbers of cancers detected incidentally by new imaging techniques and improvement in diagnosis. Although the incidence of renal cell cancer in Japan is lower than the rates in other developed countries, there is no doubt that the incidence has been increasing in recent years in Japan.^6^ The incidence rates (persons per 100,000) were 7.1 for men and 3.1 for women in 1997^16^ while they were 8.2 for men and 3.6 for women in 2002.^17^ Compared with women, men have a higher risk of renal cell cancer (age-adjusted hazard ratio = 4.52, 95% confidence interval: 2.28 to 8.96)^15^ and the risk increases with age (sex-adjusted HR = 1.08 per 10-year increment, 95% CI: 1.05 to 1.11).^15^

## Risk Factors

### Smoking and drinking

#### Tobacco

Smoking^3^,^18^–^20^ has been reported to be a risk factor for renal cell cancer. Active smoking is implicated in the etiology of renal cell carcinoma, with a strong dose-dependent in risk associated with number of cigarettes smoked per day.^19^ Furthermore, passive smoking also increased the risk of renal cell carcinoma.^20^ However, in our cohort study,^12^ smoking (current smokers vs. never smokers: age- and sex-adjusted HR = 2.13, 95% CI: 0.87 to 5.24) shows only a marginally increased risk of dying from renal cell cancer in a Japanese population because of limited statistical power due to a small number of renal cancer death in our study.

#### Alcohol

Alcohol drinking has been suggested to reduce the risk in some studies.^3^,^20^–^22^ However, in our study,^13^ there is no meaningful association between renal cell cancer death and alcohol drinking in a Japanese population.

### Dietary habits

#### Coffee and tea

No association has been found between drinking either coffee or tea and renal cell cancer in Western countries despite of numerous studies.^13^ However, a pooled analysis of 13 prospective studies suggested that coffee and tea consumption may reduce the risk of renal cell cancer.^23^ In contrast, in our study,^13^ those who drink black tea (3 +cups/day vs. none: age- and sex-adjusted HR = 13.60, 95% CI: 1.83 to 101.30) have an increased risk of renal cell cancer death even after adjusting for other factors. In addition, those who drink coffee (3 +cups/day vs. none: age- and sex-adjusted HR = 2.69, 95% CI: 0.89 to 8.10) have a marginally increased risk while there is no meaningful association between renal cell cancer death and green tea consumption in a Japanese population.^13^ In Japan, drinking black tea or coffee may be a surrogate for westernized dietary habits and thus it may be the latter rather than the former that is actually responsible for renal cell cancer. Further studies are needed to ascertain whether there is any truth to this hypothesis.

#### Foods

An inverse association between consumption of vegetables and/or fruit and risk of renal cell cancer has been seen in some studies^3^,^24^,^25^ while high intake of meat,^26^ and beef 27 have been suggested to increase the risk. Handa et al^7^ reported that both a ‘dessert’ diet factor and a ‘beef’ diet factor were associated with an increased risk of renal cell cancer, suggesting that high-fat and high-protein diets as well as sugar-and fat-rich confectioneries might be risk factors for renal cell cancer. In our study,^13^ a fondness for fatty food (age- and sex-adjusted HR = 2.64, 95% CI: 1.03 to 6.78) is associated with an increased risk of renal cell cancer while consumption of beef shows a marginally increased risk (1–2 +/week vs. seldom: age- and sex-adjusted HR = 1.73, 95% CI: 0.74 to 4.08). Since the incidence of renal cell cancer is higher in Japanese Americans than in native Japanese^5^ and it is increasing in Japan now,^6^ we cannot deny that westernization of dietary habits may play some role in the increased incidence of renal cell cancer in Japan. Chow et al^28^ also reported that an intake of staple food (i.e. bread, cereals, potatoes, rice, and spaghetti) was associated with an increased risk of renal cell cancer. On the other hand, Mucci et al^29^ reported that none of potato, bread and cereal was a risk factor for renal cell cancer. In our study,^13^ an intake of starchy roots (i.e. taro, sweet potato and potato) (3–4 +/week vs. 1–2/month or less: age- and sex-adjusted HR = 0.44, 95% CI: 0.21 to 0.94) was associated with a decreased risk of dying from renal cell cancer in a Japanese population while there is no meaningful association between renal cell cancer risk and consumption of fruits or leaf vegetables in our study.^13^ Taro^30^,^31^ and sweet potato,^32^ a part of the traditional Japanese diet, are reported to have cancer preventive potential, suggesting that these traditional diets may partly be the reasons for the lower incidence of kidney cancer death in Japan compared with the other developed countries.

### Obesity and physical activity

#### Obesity

Obesity is an established risk factor for renal cell cancer in Western countries.^1^–^3^,^18^,^33^,^34^ Insulin resistance, which is common in obesity,^35^ contributes a risk factor for numerous cancers.^36^ In a Japanese population, however, obesity shows any meaningful association with renal cell cancer death^13^ although obesity (age- and sex-adjusted HR = 1.69, 95% CI: 0.87 to 3.30) shows a marginal increased risk of dying from renal cell cancer after excluding those with a medical history of diabetes mellitus.^14^ These findings may be partly explained by the possibility that patients with diabetes mellitus are not necessarily obese in Japan because Japanese patients with diabetes mellitus may have reduce their weight after consulting with doctors for this disease as described in a Japanese textbook on diabetes mellitus.^37^

#### Physical activity

Low physical activity is a risk factor for renal cell cancer.^3^ Either occupational physical activity or leisure time physical activity is associated with an increased risk of renal cell cancer.^3^ However, our study failed to show any meaningful association between renal cell cancer risk and either occupational or leisure time physical activity in a Japanese population.^13^

### Medical conditions

#### Hypertension

Many studies have demonstrated that hypertension was associated with an increased risk of renal cell cancer in Western countries.^1^–^3^,^33^,^34^,^38^ It is difficult to distinguish the roles by hypertension and treatment of hypertension in the development of renal cell cancer because of the high correlation between them. In a Japanese population, there is a positive association between hypertension and the development of renal cell cancer (age- and sex-adjusted HR = 4.27, 95% CI: 2.07 to 8.79).^15^ In addition, hypertension is a risk factor for death from renal cell cancer (age- and sex-adjusted HR = 1.98, 95% CI: 1.06 to 3.70) in a Japanese population as well.^13^

#### Diabetes mellitus

Diabetes mellitus increases the risk of renal cell cancer in some cohort studies in Northern Europe,^39^,^40^ but it is not an established risk factor in Western countries.^1^–^3^ In a Japanese population, diabetes mellitus (age- and sex-adjusted HR = 1.72, 95% CI: 0.51 to 5.79) fails to be a significant risk factor for the development of renal cell cancer in one study^15^ while diabetes mellitus (age- and sex-adjusted HR = 2.22, 95% CI: 1.04 to 4.70) shows an increased risk of dying from renal cell cancer in another study.^14^ In addition, diabetes mellitus (age- and sex-adjusted HR = 2.59, 95% CI: 1.19 to 5.64) shows a higher risk of death from renal cell cancer after excluding obese subjects without diabetes mellitus.^14^

#### Kidney diseases

Kidney infection, kidney stone, kidney cyst, and end-stage renal disease are reported to be risk factors for renal cell cancer.^1^–^3^ In our studies, kidney disease (age- and sex-adjusted HR = 4.42, 95% CI: 1.68 to 11.63) shows an increased risk of the development of renal cell cancer in a Japanese population in one study,^15^ but kidney disease fails to be a significant risk factor for dying from renal cell cancer (age- and sex-adjusted HR = 2.35, 95% CI: 0.83 to 6.64) in another study.^13^

## Conclusion

The incidence of renal cell cancer is low in Japan.^16^,^17^ Therefore, the number of renal cancer cases was small in our studies^13^–^15^ in spite of a large population-based cohort study in Japan (JACC study).^11^ However, there is no doubt that the incidence has been increasing in recent years in Japan.^6^ The increasing incidence of renal cell cancer in a Japanese population may be partly due to westernization of the lifestyle. Our studies^13^–^15^ suggest five risk factors (i.e. hypertension, diabetes mellitus, kidney diseases, fondness for fatty food and black tea) and one preventive factor (i.e. starchy roots such as taro, sweet potato and potato) in a Japanese population. In Japan, however, drinking black tea may be a surrogate for westernized dietary habits while eating starchy roots may be a surrogate for traditional Japanese dietary habits.

The advantage of our study was a large scale prospective study among the Japanese population.^11^ However, we had limited potential to evaluate the risk of renal cell cancer because of small number of cases. Further studies may be needed to evaluate risk factors for renal cell cancer.
